# A comprehensive bedside chest radiography dataset with structured, itemized and graded radiologic reports

**DOI:** 10.1038/s41597-026-07271-7

**Published:** 2026-04-21

**Authors:** Daniel Truhn, Daniel Geiger, Robert Siepmann, Marc Sebastian von der Stück, Keno Kyrill Bressem, Jakob Nikolas Kather, Christiane Kuhl, Gustav Müller-Franzes, Sven Nebelung

**Affiliations:** 1 Department of Diagnostic and Interventional Radiology, University Hospital RWTH Aachen, Aachen, Germany; 2 Department of Diagnostic and Interventional Radiology, Technical University of Munich, School of Medicine and Health, Klinikum rechts der Isar, TUM University Hospital, Munich, Germany; 3 Department of Cardiovascular Radiology and Nuclear Medicine, Technical University of Munich, School of Medicine and Health, German Heart Center, TUM University Hospital, Munich, Germany; 4 Else Kroener Fresenius Center for Digital Health, University Hospital Carl Gustav Carus, Dresden, Germany; 5 Technical University Dresden, Dresden, Germany

**Keywords:** Medical research, Radiography

## Abstract

Automated analysis of bedside chest radiographs remains challenging due to limited large-scale datasets with expert annotations and standardized severity grading. We provide TAIX-Ray, a comprehensive dataset of 215,381 bedside chest radiographs collected from 47,724 intensive care unit patients (30,306 male, 17,418 female, median age 68 years) collected over 14 years (01/2010-12/2023) at the University Hospital Aachen, Germany. During routine clinical reporting, 134 trained radiologists provided structured, itemized reports using a standardized template. They systematically assessed eight pathological findings: heart size (cardiomegaly), pulmonary congestion, pleural effusion (left/right), pulmonary opacities (left/right), and atelectasis (left/right) using a five-point ordinal severity scale (absent, questionable, mild, moderate, severe). The dataset includes (i) bedside chest radiographs (anteroposterior projections), (ii) structured, itemized reports, (iii) patient demographics (age and sex), and (iv) the temporal metadata. To facilitate immediate research adoption, we provide a baseline transformer model, implementation code, and predefined data splits, ensuring reproducible benchmarking. This resource enables the development of clinical AI models for automated pathology detection and severity assessment in critical care settings.

## Background & Summary

Intensive care units rely heavily on bedside chest radiography for monitoring critically ill patients, yet interpreting these images presents unique challenges due to suboptimal positioning, patient-related artifacts, and the complex interplay of multiple pathologies often present simultaneously^1^. Still, timely diagnosis is important for patient treatment, and automated interpretation systems could provide valuable support for radiologic assessment in this clinical setting, potentially improving diagnostic accuracy and workflow efficiency^2–4^.

Current large-scale chest radiography datasets have enabled substantial advances in deep learning applications, yet they suffer from fundamental limitations in label quality that constrain model performance. Datasets such as ChestX-ray14^5^ (112,120 radiographs), ReXGradient-160K^6^ (160,000 radiographs), PadChest^7^ (168,861 radiographs), CheXpert^8^ (224,316 radiographs), and MIMIC-CXR^9^ (371,920 radiographs) rely primarily on natural language processing (NLP) extraction from free-text reports to generate labels. However, NLP approaches face inherent challenges with ambiguous terms that can be interpreted differently, depending on context, incorrect use of grammar in fast-paced clinical environments, and the difficulty of capturing nuanced expressions of diagnostic uncertainty^10^. These limitations are compounded by substantial variability among radiologists in how they express uncertainty, with 55% of thoracic imaging examinations containing at least one expression of uncertainty^11^. Consequently, NLP methods may fail to capture the fine granularities of clinical communication as intended by the radiologist^12–14^. Even if supplemented with additional categories such as “uncertain” and validation and correction techniques, the post-hoc binarization of “disease present” vs. “disease absent” may only partially reflect the intended clinical communication of the original radiologic report. This label noise likely affects the performance of deep learning models trained on these datasets. As demonstrated by Sjoding *et al*., who trained a convolutional neural network (CNN) to detect acute respiratory distress syndrome (ARDS), reducing label noise improved the area under the receiver operating characteristic curve (AUROC) from 0.88 to 0.93^15^. Similarly, Dunnmon *et al*. used a dataset of 216,431 chest radiographs, of which 10% were newly annotated by a radiologist. Even using only 10% of the original dataset size, they were able to train a CNN that outperformed the model trained on the full dataset with NLP-generated labels^16^.

These issues can be addressed with labels from prospective, structured annotations created during routine clinical reporting. When radiologists assess images with full clinical context (including patient history, laboratory parameters, and concurrent imaging), they provide more informed and clinically relevant interpretations than post-hoc NLP extraction can capture. Furthermore, severity grading and anatomic localization captured at the time of reporting preserve clinical nuances that binary presence/absence labels cannot convey.

We present TAIX-Ray, a dataset of 215,381 bedside chest radiographs from intensive care patients with structured, prospective annotations provided by 134 radiologists during routine clinical reporting over 14 years. Unlike existing datasets that rely on post-hoc NLP extraction, these annotations were generated using standardized templates that capture the presence and severity of seven key pathological findings on a five-point ordinal scale. This approach addresses the core limitations of current datasets while reflecting real-world clinical workflows and decision-making processes. Previous work using subsets of this dataset has demonstrated superior diagnostic agreement with expert panels compared to individual radiologists^3^, successful integration of imaging and clinical data^17^, and robust performance across various analytical approaches, ranging from basic statistics to advanced ML-based predictive modeling^18^.

By providing the full dataset alongside baseline models, code, and standardized data splits, we aim to facilitate further research in automated chest radiography interpretation and to establish new benchmarks for label quality and clinical relevance.

## Methods

### Data collection

We retrospectively collected 215,381 bedside chest radiographs from 47,724 intensive care unit (ICU) patients at the University Hospital Aachen, Germany, spanning 14 years from January 2010 to December 2023. Radiographs were acquired across 10 different ICU wards serving all clinical disciplines using 18 mobile radiography systems (Mobilett MIRA; Siemens Healthineers).

All examinations were performed by trained radiologic technologists as anteroposterior projections with automatic exposure control. Conventional screen-film systems were used until 2016, when digital flat-panel detectors were introduced. Patient positioning and source-to-image distances varied because the images were acquired at the bedside.

The study was approved by the Ethics Committee of the RWTH Aachen Faculty of Medicine on March 7th, 2025 (ID EK 25-080). The study procedures were carried out in accordance with the relevant ethical guidelines and regulations. The need for obtaining informed patient consent was waived because all patient-identifiable information in the data has been removed.

### Radiologist annotations

Reports were provided by radiologists who were assigned to the radiography workplace. These radiologists had completed specialist training in radiography interpretation (equivalent to board-certification level) or were board-certified. If radiologists had undergone neither specialist training nor board certification, their reports were validated by board-certified radiologists. The identifier of the final validating radiologist is provided for this dataset.

The radiographs were read using the in-house picture archiving and communication system (iSite, Philips Healthcare) operated on dedicated radiology workstations, with reporting monitors and diagnostic displays set at calibrated luminance and controlled lighting conditions. Reporting followed institutional guidelines and standard radiologic practice, as defined by the Fleischner Society and other professional organizations^19,20^. During the reporting process, radiologists had access to comprehensive clinical information, including prior imaging studies, cross-sectional imaging, laboratory values, and functional test results.

A total of 134 radiologists participated in reading and interpreting the radiographs, as part of their clinical routine reporting duties. Overall, each radiologist provided an average of 1,607 reports. All reports were generated using a structured reporting template integrated into the radiology information system. This template was designed to facilitate efficient, precise, and detailed documentation of imaging findings and served as the department’s default reporting mode for bedside chest radiographs. Consequently, each radiograph in the dataset was annotated by a single reporting radiologist as part of routine clinical care, and no radiograph was independently annotated by more than one reader.

The structured template assessed five key imaging findings: heart size, pulmonary congestion, left/right pleural effusion, left/right atelectasis, and left/right pulmonary opacities. For most findings, presence and severity were graded using a standardized five-point scale ranging from ‘none’ (‘-‘), ‘questionable’ (‘(+)’) to ‘mildly present’ (‘+’), ‘moderately present’ (‘++’), to ‘strongly present’ (‘+++’) (Table 1). Heart size was assessed using a distinct categorical scale: ‘normal’, ‘borderline enlarged’, ‘enlarged’, ‘massively enlarged’, and ‘not assessable’.

**Table 1:** Details of Itemized Reporting Template Used for Prospective Labeling of Bedside Chest Radiography During Clinical Reporting and Encoding Details.

Imaging Finding	Grading	Encoding in CSV
**Heart Size**	Normal	0
	Borderline Enlarged	1
	Enlarged	2
	Massively Enlarged	3
**Pulmonary Congestion**	None	0
	Questionable	1
	Mild	2
	Moderate	3
	Severe	4
Pleural Effusion (*)	None	0
	Questionable	1
	Mild	2
	Moderate	3
	Severe	4
Pulmonary Opacities (*)	None	0
	Questionable	1
	Mild	2
	Moderate	3
	Severe	4
Atelectasis (*)	None	0
	Questionable	1
	Mild	2
	Moderate	3
	Severe	4

The clinical reporting framework operated under the assumption of linear associations with equal intervals between adjacent scale points, such that the difference between ‘none’ (‘-’) and ‘questionable’ (‘+’) was considered the same as between ‘moderately present’ (‘++’) and ‘strongly present’ (‘+++’).

Imaging findings were graded based on severity and/or distribution, as indicated by the detailed encodings. For ‘heart size’, the structured reporting template included the option ‘not assessable’, coded as ‘−1’; however, this option was not selected in any report and therefore does not occur in the dataset. (*) denotes separate labels for the right and left chest.

### Anonymization

All personally identifiable information was systematically removed from the radiographs and radiologic reports in accordance with local data protection regulations. Radiologist identifiers were replaced with unique anonymized codes. To preserve temporal relationships, acquisition timestamps were retained, with temporal obfuscation applied. Radiograph’s acquisition date was randomly shifted by a uniformly distributed offset ranging from −100 to +100 days.

### Image preprocessing

All radiographs were converted from DICOM to 16-bit grayscale PNG format using a standardized and fully reproducible preprocessing pipeline to preserve the full dynamic range and subtle intensity variations of the original images. During DICOM conversion, pixel data were extracted and inspected for modality-related intensity transformations. Specifically, the presence of modality-level rescaling was evaluated via the *Modality LUT* and the *Rescale Slope* and *Rescale Intercept* DICOM tags. No ModalityLUTSequence was present in the dataset, and all images exhibited RescaleSlope values of 1 (or NaN) and RescaleIntercept values of 0 (or NaN). Under these conditions, applying modality-level intensity rescaling would not alter pixel values; therefore, no such transformation was applied.

Photometric interpretation was handled explicitly, with images stored as MONOCHROME2 after inversion where required. VOI LUT-based windowing was intentionally not applied, as VOI LUTs are primarily designed for display optimization in clinical viewing environments and are often manufacturer- and modality-specific. Preserving the native, manufacturer-independent pixel-intensity representation was therefore preferred to support reproducible downstream machine-learning analyses.

No global intensity normalization or dynamic range standardization was performed. The exported PNG images intentionally preserve the native intensity ranges of the original DICOM files, which may vary due to acquisition hardware, bit depth, and manufacturer-specific scaling. This design choice retains acquisition-dependent information and allows downstream users to apply task-specific normalization strategies tailored to their machine learning applications.

Subsequently, each image was uniformly resized such that its longer dimension was standardized to 512 pixels while maintaining the original aspect ratio. Resizing was performed using bilinear interpolation. For completeness, we also provide the radiographs at their original resolution alongside the standardized 512-pixel versions.

### Annotation preprocessing

Although radiologists used the itemized structured reporting template, the completed forms were automatically converted to plain text to ensure compatibility with the hospital information system. During reporting, radiologists had the option to add additional narrative comments, such as “Progression of itemized imaging finding X compared to the previous study”, or other contextual information to the individual item fields.

For data curation and to prevent the inclusion of potentially patient-identifiable information, all textual content beyond the predefined grading options was systematically parsed using a customized parser that extracted only standardized classification terms.

This parser used regular expressions specifically tailored to identify and extract the predefined grading terminology. For “Heart Size” assessments, recognized terms such as “normal”, “borderline enlarged”, “enlarged”, and “massively enlarged” were mapped to numerical values (Table 1). The parser incorporated grammatical variations of the terms to ensure comprehensive and accurate classification.

For the remaining classes, severity gradings (“none”, “(+)”, “+“, “++“, “+++”) were identified and converted into structured numeric labels. Bilateral findings, such as “Pleural Effusion” and “Pulmonary Opacities,” required laterality-specific parsing. The parser identified expressions formatted as “re < GRADING > ; li < GRADING > “, where GRADING represents one of the predefined grading severity terms, and separated these into distinct right and left assessments.

This preprocessing approach ensured that only standardized, predefined grading options remained in the dataset, while additional narrative comments or comparisons with previous studies were excluded.

A total of 1,281 studies with missing required information (e.g., date of birth, sex, report, or study date) and 28,163 studies lacking associated radiographs were excluded. Additionally, if multiple radiographs were present within the same study series and it was unclear which specific radiograph the radiologists used for the final assessment, the entire study was removed.

### Data splits

The dataset was partitioned into training, validation, and test sets at the patient level to ensure robust, reproducible model evaluation and benchmarking. Patients were uniquely assigned to a single set to prevent data leakage, i.e., radiographs of the same patient appearing in different sets.

## Data Record

The TAIX-Ray dataset is available at HuggingFace^21^ (https://huggingface.co/datasets/TLAIM/TAIX-Ray). It comprises three main components: (i) the bedside chest radiographs, (ii) the corresponding itemized and structured annotations, and (iii) the split configuration.

### Image files

All radiographs were stored in the “images” directory as 16-bit grayscale PNG files. Each radiograph was named using a unique identifier: “$UID.png,” corresponding to a specific radiograph in the dataset. The unique identifier links the radiographs to their annotations.

### Annotation file

**Fig. 1:**
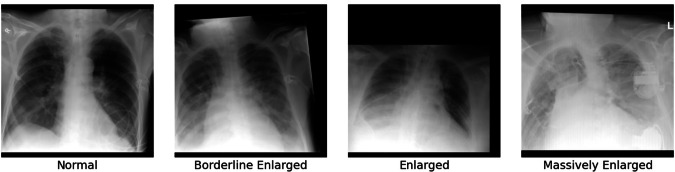
Example Radiographs with Different Labels for “Heart Size”. Heart size was graded as normal, borderline enlarged, enlarged, or massively enlarged. In clinical contexts, enlarged heart size is usually referred to as “cardiomegaly”.
- **PulmonaryCongestion**: Severity grading for pulmonary congestion, encoded as per Table 1. Examples of the different grades are given in Fig. 2.

The annotations are provided in a CSV file named “annotation.csv.” This file contains structured information for each radiograph and includes the following columns:

- **UID**: The unique identifier (256-bit hash) of the radiograph, matching the filename without the.png extension.
- **PatientID**: A unique identifier (256-bit hash) for each patient to ensure patient-level anonymity while allowing for patient-specific analyses if required.
- **PhysicianID**: A unique identifier (256-bit hash) for each radiologist who read the radiograph during clinical reporting and, thus, performed the annotation.
- **Age**: Patient age at the time of radiograph acquisition in days.
- **Sex**: Patient sex, either “M” for male or “F” for female.
- **StudyDate**: Temporally obfuscated acquisition date designed to preserve patient privacy while maintaining analytical utility. Rather than providing the exact acquisition timestamps, a random offset ranging from −100 to 100 days was applied. This offset is consistent across all radiographs for the same patient but varies between patients. Dates are formatted according to the ISO 8601 standard (YYYY-MM-DD).
- **HeartSize**: Severity grading for heart size, encoded as per Table 1. An example of the different grades is given in Fig. 1.

**Fig. 2:**
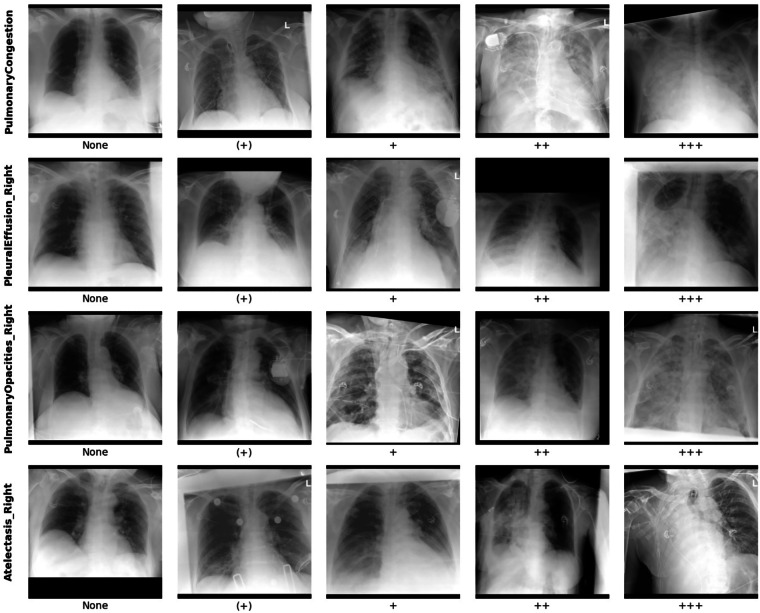
Example Radiographs with Different Labels for “Pulmonary Congestion”, “Pleural Effusion”, “Pulmonary Opacities”, and “Atelectasis”.
- **PleuralEffusion_Left**: Severity grading for pleural effusion on the left (patient) side, encoded as per Table 1.
- **PleuralEffusion_Right**: Severity grading for pleural effusion on the right (patient) side, encoded as per Table 1. Examples of the different grades are given in Fig. 2.
- **PulmonaryOpacities_Left**: Severity grading for pulmonary opacities on the left (patient) side, encoded as per Table 1.
- **PulmonaryOpacities_Right**: Severity grading for pulmonary opacities on the right (patient) side, encoded as per Table 1. Examples of the different grades are given in Fig. 2.
- **Atelectasis_Left**: Severity grading for atelectasis on the left (patient) side, encoded as per Table 1.
- **Atelectasis_Right**: Severity grading for atelectasis on the right (patient) side, encoded as per Table 1. Examples of the different grades are given in Fig. 2.

Label categories as provided by the reporting radiologists. Apart from “pulmonary congestion”, only right-sided pathologies are visualized to avoid redundancy.

### Split file

To facilitate standardized model development and evaluation for automated detection and quantification of imaging findings, the dataset contains predefined partitioning information. As detailed above, the radiographs and annotations were partitioned into predefined training, validation, and test sets, with no patient overlap. The partition is provided in a CSV file named “split.csv” with the following columns:

- **UID**: The unique identifier as defined in the annotation file.
- **Split**: String that separates into the “train” (training), “val” (validation), and “test” sets.

## Data Overview

The final dataset included 215,381 radiographs from 47,724 patients. The cohort had a mean age of 66 ± 15 years (range, 0–110), with 36% women (n = 17,418). The data were divided into a training set of 137,593 radiographs from 30,543 patients (64%), a validation set of 34,860 radiographs from 7,636 patients (16%), and a test set of 42,928 radiographs from 9,545 patients (20%) (Table 2).

**Table 2:** Characteristics of the Training, Validation, and Test Sets.

	Training	Validation	Test
**Radiographs [n (%)]**	137,593 (63.9%)	34,860 (16.2%)	42,928 (19.9%)
**Patients [n (%)]**	30,543 (64.0%)	7,636 (16.0%)	9,545 (20.0%)
**Age [median – 95% interval (years)]**	68 [29–87]	69 [30–86]	68 [27–87]
**Sex [n (%)]**			
**Male**	19,402 (63.5%)	4,837 (63.3%)	6,067 (63.6%)
**Female**	11,141 (36.5%)	2,799 (36.7%)	3,478 (36.4%)

**Fig. 3:**
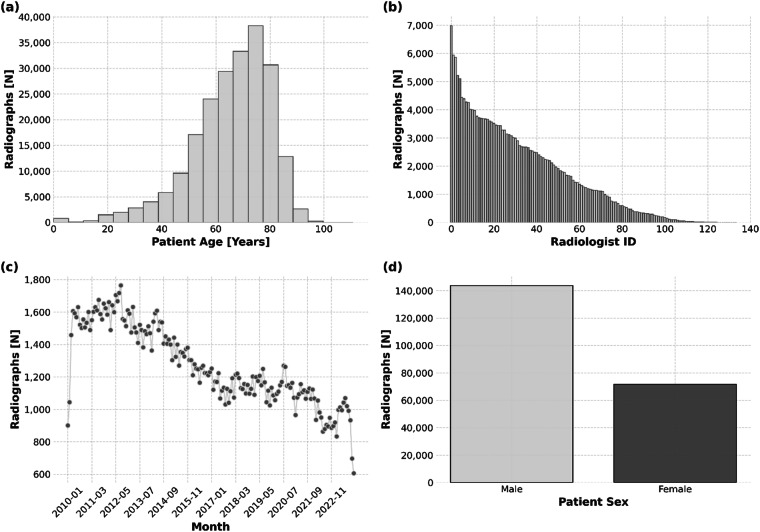
Dataset Characteristics and Distribution. (**a**) Patient age distribution. The x-axis is scaled in 5-year intervals. (**b**) Number of radiographs contributed by individual radiologists (n = 134). (**c**) Number of radiographs collected per month from January 2010 to December 2023. (**d**) Radiograph distribution by patient sex.

The frequency distributions for all assessed imaging findings are provided in Table 3. The frequency distributions of radiographs across different age groups, radiologists, acquisition dates, and patient sex are illustrated in Fig. 3.

**Table 3:** Frequency Counts of the Severity Gradings as a Function of Imaging Finding.

Imaging Finding	None	Questionable	Mild	Moderate	Severe
**Pulmonary Congestion**	73076 (33.9%)	24088 (11.2%)	97861 (45.4%)	19650 (9.1%)	706 (0.3%)
**Pleural Effusion (Left)**	76117 (35.3%)	30935 (14.4%)	93884 (43.6%)	14040 (6.5%)	405 (0.2%)
**Pleural Effusion (Right)**	107319 (49.8%)	24347 (11.3%)	65372 (30.4%)	17916 (8.3%)	427 (0.2%)
**Pulmonary Opacities (Left)**	125031 (58.1%)	20446 (9.5%)	49322 (22.9%)	18061 (8.4%)	2521 (1.2%)
**Pulmonary Opacities (Right)**	106376 (49.4%)	21586 (10.0%)	58588 (27.2%)	25266 (11.7%)	3565 (1.7%)
**Atelectasis (Left)**	50041 (23.2%)	15114 (7.0%)	131986 (61.3%)	17370 (8.1%)	870 (0.4%)
**Atelectasis (Right)**	57035 (26.5%)	14025 (6.5%)	120356 (55.9%)	22859 (10.6%)	1106 (0.5%)
	**Normal**	**Borderline Enlarged**	**Enlarged**	**Massively Enlarged**	
**Heart Size**	74352 (34.5%)	30797 (14.3%)	100488 (46.7%)	9744 (4.5%)	

Indicated are the frequencies and percentages for each severity level across the different imaging findings, presented as “n (%).”

## Technical Validation

### Baseline model development

To establish a benchmark for automated detection and grading of imaging findings in bedside chest radiographs, we developed a baseline model using a Vision Transformer architecture and DINOv2 pre-training^22^. DINOv2 is a state-of-the-art self-supervised learning framework that enables efficient pretraining of Vision Transformers on large-scale datasets without labeled data. The pretrained Transformer, initialized with DINOv2 weights, serves as a robust feature extracting backbone^23^. We added a custom classification head to predict the presence and severity of the predefined imaging findings.

The model was fine-tuned on our dataset to perform two classification tasks:

1. Binary Classification (normal vs. abnormal):
  In the first experiment, the model was trained to determine whether each radiograph was normal (i.e., absence of a pathologic imaging finding) or abnormal (i.e., presence of a pathologic imaging finding, regardless of grade). This setup assesses the model’s screening ability, aligning with most published models that lack grading information^16,24^.
2. Ordinal Classification:
  In the second experiment, the model was trained to classify each imaging finding based on graded severity or distribution (Table 1). This setup evaluates the model’s ability to detect a pathologic imaging finding and quantify its severity or distribution.

The training involved resizing the radiographs to a uniform resolution suitable for the Transformer architecture (448 × 448), applying intensity normalization, and employing data augmentation, i.e., random rotations and vertical flips. We used binary cross-entropy loss for the binary classification task and the CORN (Conditional Ordinal Regression for Neural Networks)^25^ loss for the multiclass severity classification task, accounting for the ordinal nature of graded severity or distribution.

Optimization was performed using the AdamW optimizer with an initial learning rate of 1e-6 and weight decay of 1e-2. The training was conducted with a batch size of 24, using early stopping based on validation performance to prevent overfitting.

We evaluated model performance on the test set using classification accuracy for both tasks. For binary classification, we calculated the AUROC for each imaging finding.

For multiclass (ordinal) classification of severity or distribution, we computed linear weighted Cohen’s kappa coefficients to assess agreement with each radiologist’s original annotation.

The model was implemented using PyTorch (version 2.6) and trained on a single NVIDIA L40S GPU for about 11 hours. For reproducibility, we provide training and evaluation scripts in the code repository, including detailed code for the Transformer architecture and custom classification heads. Consequently, the repository includes the complete codebase with documentation, fine-tuned model weights, predefined training, validation, and test splits.

### Performance of baseline models

**Fig. 4:**
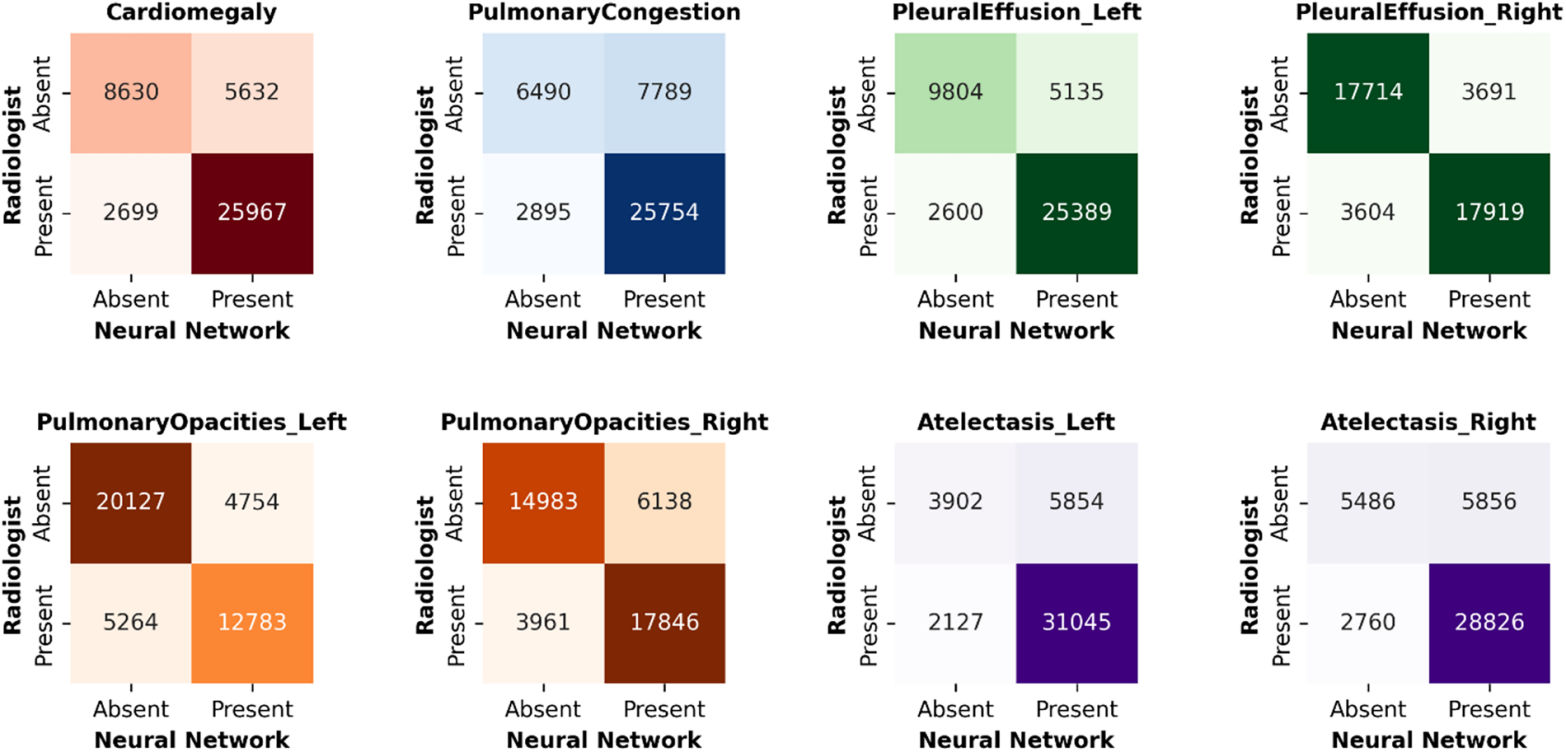
Confusion Matrices for Binary Classification as a Function of Imaging Finding. Indicated are the numbers of radiographs with an imaging finding present (for ‘heart size’ [’cardiomegaly’]: ‘borderline enlarged’, ‘enlarged’, ‘massively enlarged’; for the other conditions: ‘questionable’, ‘mild’, ‘moderate’, ‘severe’) or absent (for ‘heart size’ [’cardiomegaly’]: ‘normal’; for the other conditions: ‘none’), respectively.

**Fig. 5:**
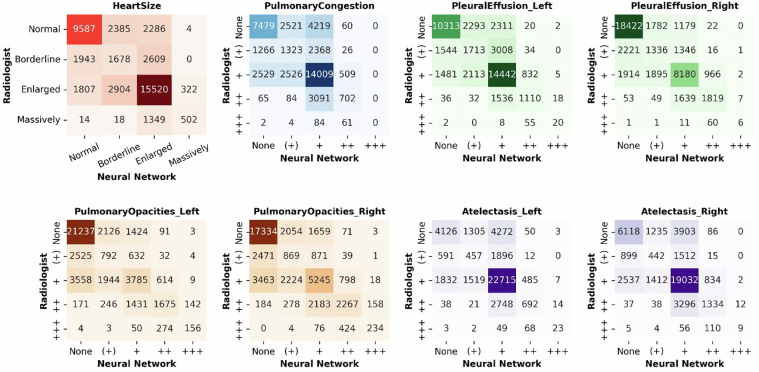
Confusion Matrices for Multiclass (Ordinal) Classification as a Function of Imaging Finding.

The baseline models’ performance metrics are provided in Table 4, while Figs. 4 and 5 detail the corresponding confusion matrices.

**Table 4:** Performance Metrics for the Binary and Ordinal Classification Models.

	Binary Classification		Ordinal Classification	
	AUROC	Accuracy	Cohen’s κ	Accuracy
**Heart Size**	0.87 [0.86–0.87]	0.81 [0.80–0.81]	0.54 [0.53–0.55]	0.64 [0.63–0.64]
**Pulmonary Congestion**	0.80 [0.79–0.80]	0.75 [0.75–0.76]	0.40 [0.39–0.41]	0.55 [0.54–0.55]
**Pleural Effusion (Left)**	0.89 [0.89–0.89]	0.82 [0.82–0.82]	0.57 [0.57–0.58]	0.64 [0.64–0.65]
**Pleural Effusion (Right)**	0.91 [0.91–0.92]	0.83 [0.83–0.83]	0.65 [0.65–0.66]	0.69 [0.69–0.70]
**Pulmonary Opacities (Left)**	0.84 [0.84–0.85]	0.77 [0.76–0.77]	0.53 [0.53–0.54]	0.64 [0.64–0.65]
**Pulmonary Opacities (Right)**	0.85 [0.85–0.86]	0.76 [0.76–0.77]	0.55 [0.55–0.56]	0.60 [0.60–0.61]
**Atelectasis (Left)**	0.81 [0.81–0.82]	0.81 [0.81–0.82]	0.39 [0.38–0.40]	0.65 [0.65–0.66]
**Atelectasis (Right)**	0.83 [0.83–0.84]	0.80 [0.80–0.80]	0.45 [0.44–0.46]	0.63 [0.62–0.63]

Performance is reported in terms of Area Under the Receiver Operating Characteristic Curve (AUROC), Accuracy, and linear weighted Cohen’s κ (versus radiologists’ original reports). Results are presented as point estimates with 95% confidence intervals, derived through bootstrapping with 1000 resamples.

Figure organization as in Fig. 4. Labels as defined in Table 1.

## Data Availability

The TAIX-Ray dataset generated and analyzed during the current study is publicly available on at Hugging Face: https://huggingface.co/datasets/TLAIM/TAIX-Ray. The dataset includes bedside chest radiographs, structured annotations, and predefined training, validation, and test splits.

## Code availability

The code used for baseline model development and evaluation is available at https://github.com/TruhnLab/TAIX-Ray.
